# Cold-induced urticaria after black ant bite anaphylaxis

**DOI:** 10.5339/qmj.2023.sqac.5

**Published:** 2023-11-19

**Authors:** Sara Mohamed, Hassan Mobayed

**Affiliations:** ^1^Allergy & Clinical Immunology Division, Department of Medicine, Hamad Medical Corporation, Doha, Qatar Email: SMohamed54@hamad.qa

## Abstract

Introduction: Acquired cold-induced urticaria is a form of physical urticaria that is usually spontaneous. However, reports have shown that bees, wasps, or jellyfish stings can trigger it. We report the first case of cold-induced urticaria following black ant bite-induced anaphylaxis.

Case Report: A 41-year-old lady with no chronic illness with a known black ant bite allergy history. Three years ago, she sustained a black ant bite that required an emergency room visit to treat anaphylaxis. A few days later, she developed attacks of generalized hives on exposure to cold air and objects. She was started on desloratadine tablets which controlled her symptoms. The patient was given EpiPen and instructed to avoid black ants’ approach and exposure to cold. She was then followed up in our clinic.

Discussions and Conclusions: Acquired cold-induced urticaria is a form of physical chronic inducible urticaria. Physical urticarias account for 25 % of chronic urticarias. The patient can have wheals, angioedema, or both in response to the cold exposure. Symptoms can be mild or severe, limiting the patient’s quality of life. Acquired cold urticaria is idiopathic; however, cases have been reported after different triggers, such as insect stings (bees, wasps, and jellyfish). The black Samsum ant is a recognized trigger of allergic reactions in Qatar and the Gulf region. In a study done in Qatar, 23.5% of anaphylaxis cases were due to black ant stings. There are no validated or standardized skin tests or immunotherapy for the black Samsum ant, which necessitates physicians to be careful in assessing such patients and focus on taking a detailed history. The limitation of testing and immune therapy makes history the tool for diagnosis, and avoidance is the mainstay of treatment.
